# The Influence of Incorporating Essential Plant Oils in the Lactic Fermentation Process of Cabbage on the Quality of Sauerkraut

**DOI:** 10.3390/foods15152746

**Published:** 2026-08-05

**Authors:** Felicia Tuțulescu, Mira Elena Ionică, Felicia Stoica

**Affiliations:** Department of Horticulture & Food Science, Faculty of Horticulture, University of Craiova, A.I. Cuza Street, No. 13, 200585 Craiova, Romania; mira.ionica@edu.ucv.ro (M.E.I.); felicia.stoica@edu.ucv.ro (F.S.)

**Keywords:** sauerkraut, lactic acid bacteria, essential oils

## Abstract

Cabbage (*Brassica oleracea* var. *capitata*) is considered a healthy vegetable due to its chemical composition and high nutritional value, owing to its main constituents of carbohydrates and dietary fiber and the fact that it is also a source of ascorbic acid. The resulting product, sauerkraut, is low-calorie and has a long shelf life. Lactic acid bacteria play the most important role in cabbage fermentation, and their activity is influenced by physical factors such as temperature and chemical factors such as salt concentration and the addition of spices, which may inhibit undesirable microflora, in addition to their flavoring effect. This study explores the impact of incorporating essential oils into sauerkraut production on the lactic acid bacteria that drive cabbage fermentation. It also analyzes how these oils affect the primary chemical components of sauerkraut, including total soluble solids, total phenolics, and antioxidant activity. Essential oils from thyme, dill, wild thyme, laurel, and basil are tested. Incorporating essential oils into cabbage before fermentation may partially inhibit or slow the growth of bacteria involved in the fermentation process while also contributing to the improved flavor profile of the final product.

## 1. Introduction

*Brassica* vegetables, including cabbage, are well known for their nutritional value. These vegetables contain vitamins with antioxidant properties, such as ascorbic acid, β-carotene, folic acid, and tocopherol, and mineral compounds, including calcium, selenium, potassium, magnesium, and iron, as well as antioxidants like flavonoids, phenolics, and glucosinolates [1]. Cabbage has a strong nutritional composition and is considered a beneficial vegetable for health. Even though some compounds are lost during fermentation, sauerkraut retains a high nutritional content and is enriched in valuable compounds that benefit health [2]. Sauerkraut is a fermented product widely consumed in Central and Eastern Europe [3]. It is traditionally obtained through the spontaneous fermentation of cabbage via the epiphytic microbiota [4]. Sauerkraut is a low-calorie food that can be preserved for an extended period without losing its nutritional or sensory qualities [5,6]. In addition to being rich in vitamins and minerals, sauerkraut contains elevated levels of products resulting from glucosinolate hydrolysis, which have demonstrated anti-cancer effects [7]. This process has been optimized and occurs under anaerobic conditions, with the addition of an adequate amount of salt [8], and the production and properties of cabbage are largely dependent on the presence of microbiota and fermentation conditions. Studies conducted on the optimal salt concentration required for sauerkraut have shown that it ranges between 1 and 3% [9,10]. Premakumar et al. [4] demonstrated that sauerkraut with 3% NaCl, stored at room temperature after fermentation, exhibited the best nutritional characteristics. The degree of microbial development and the sensory properties of the final product depend on the addition of sodium chloride [11]. A key role is played by lactic acid bacteria, which can produce organic acids, bacteriocins, and vitamins, as well as volatile compounds that influence the flavor of fermented foods. This flavor is further determined by components derived from the raw material, which undergo changes during fermentation (consisting mainly of glucosinolates), and compounds formed by microorganisms during lactic fermentation [6]. Inoculation with starter cultures has increasingly been used to enhance the quality of sauerkraut. Lactic acid bacteria have been widely employed in various fermented foods to shorten the fermentation period, improve their nutritional qualities, and modify the organoleptic properties of the final products. Yuwono et al. [12] conducted studies on the use of lactic acid bacterial starter cultures for the fermentation of cabbage with a lower salt content. Considering the increasing diversity of vegetable fermentations, studies have been carried out on the benefits of both allochthonous and autochthonous starter cultures for the controlled fermentation of vegetables, including cabbage [13]. The principle of cabbage fermentation is based on the selection of bacteria using sodium chloride solution at different concentrations. These bacteria convert the sugars present in cabbage into lactic acid. Lactic acid accumulates throughout several stages of fermentation, and when it reaches concentrations of 2% in the solution, it becomes a natural preservative that stabilizes the final product. Therefore, one of the most important conditions in directing the fermentation process is an environment that promotes the development of lactic acid bacteria. The preserving effect of lactic acid bacteria on food is also well established, extending the shelf life and increasing the nutritional value of the resulting final product [14]. After lactic fermentation, the bioavailability of certain micronutrients in raw cabbage increases [15]. Most lactic acid-producing bacteria are heterotrophic and generally require many growth factors for cellular growth and biosynthetic capabilities, including nucleotides, vitamins, amino acids, peptides, and fatty acids [16]. The lactic fermentation process is extremely complex, involving many types of microorganisms [17], and it can be divided into four main phases: the initial aerobic, intense fermentation, stable, and anaerobic fermentation phases [18,19,20]. Therefore, understanding the sequence of microorganisms and the correlation between microorganisms and the quality of fermentation in different fermentation phases can provide a scientific basis for modulating fermentation. Tlais et al. [21] conducted studies that provide new insight into the sequence of bacteria responsible for lactic fermentation in terms of fermentation steps and the resulting metabolic functions, which are essential for industrial sauerkraut production. There are several methods for preserving the final product (storage at low temperatures, pasteurization, addition of preservatives, etc.). During pasteurization, certain essential nutrients in cabbage, such as vitamins, are degraded. [22]. Sauerkraut can be packaged in glass jars, cans, and plastic bags. Meanwhile, unpasteurized sauerkraut is preserved using sodium benzoate (0.1%) and potassium metabisulfite as preservatives, or it can be kept refrigerated [23]. Essential oils are volatile aromatic compounds, usually extracted through steam distillation or cold pressing, which retain the scent and therapeutic properties of the parent plant. Each species has a unique profile of the essential oils it contains. Chemically, they are mixtures of organic substances, the most common being terpenes and phenols. They are distinguished by a potent antimicrobial action, particularly their antibacterial properties. The present study examined the effect of some plant extracts (essential oils) on the lactic acid bacteria responsible for the fermentation of cabbage. Extracts from wild thyme, dill, thyme, laurel, and basil were tested.

## 2. Materials and Methods

### 2.1. Reagents

Analytical-grade reagents, including sodium carbonate and methanol, were sourced from Merck (Darmstadt, Germany. The Folin–Ciocalteu reagent, Trolox (6-hydroxy-2,5,7,8-tetramethylchroman-2-carboxylic acid), DPPH (2,2-diphenyl-1-picrylhydrazyl), gallic acid, and sodium acetate were obtained from Sigma-Aldrich (Darmstadt, Germany). All other chemicals utilized in this study were of analytical purity.

### 2.2. Plant Material

The study material consisted of cabbage from the ‘*De Buzău’* variety, which is a white cabbage cultivar valued for its productivity and disease resistance. It is a late-season variety with a long growing period, thus making it highly suitable for preservation through lactic acid fermentation. The raw material was sourced and cultivated from a private producer in the Oltenia region (Saioc PFA, Scarisoara, Olt country, Romania; 43.99° N, 24.57° E).

### 2.3. Extraction of Essential Oils

The oils tested were extracted using hydrodistillation (Neo Clevenger apparatus, Labbox Deutschland GmbH, Düsseldorf, Germany). The following plant materials for the extraction of essential oils were used: dill (*Anethum graveolens*), thyme (*Satureja hortensis L.*), wild thyme (*Thymus serpyllum*), laurel (*Laurus nobilis*), and basil (*Ocimum basilicum L*.). The leaves of these species were dried and finely milled. The leaves were dried at room temperature (in the shade, with air circulation) until a constant mass was reached, in order to preserve the volatile constituents while ensuring adequate storage stability. Room-temperature drying was selected to avoid the thermal degradation and loss of essential oil that can occur at elevated drying temperatures [24]. Prior to hydrodistillation, the dried leaves were reduced to a coarsely comminuted material rather than a fine powder. For essential-oil-bearing plants, coarse comminution is preferred because excessively fine grinding can promote loss and volatilisation of volatile constituents, impede steam penetration, and cause clogging of the plant bed during hydrodistillation [25]. This is consistent with the European Pharmacopoeia monograph, which specifies the drug as whole or cut, and with reported hydrodistillation studies in which larger particle sizes yielded higher essential oil recovery [25]. Subsequently, 100 g of each species was combined with 1 L of water in the distillation flask of the Clevenger apparatus. Hydrodistillation was performed at 102 °C for 180 min. The collected oil varied in quantity depending on the species, ranging from 0.6 to 1.4 mL/100 g dry weight. Until the oils were used, they were stored in airtight bottles in a refrigerator at a temperature of 4 °C.

### 2.4. Experimental Design

Lactic fermentation was conducted at both 14 and 22 °C. For fermentation, the raw material was conditioned by sorting, cleaning. Whole cabbage heads were shredded using a conventional electric vegetable slicer/grater (Philips HR1388/80, 200 W; Philips, Bucharest, Romania) to obtain finely shredded cabbage. The shredded cabbage was placed into 5 L glass jars, each containing 2.0 kg of cabbage. An emulsified brine containing plant essential oils was then add-ed (0.015%). A total volume of 2.5 L of the solution was added to each container.

Emulsification was performed using an ultrasonic homogenizer (Hielscher UP400St, 400 W; Hielscher Ultrasonics GmbH, Teltow, Germany). A total volume of 2.5 L of the solution was added to each container.

The essential oils were selected from the list of oils approved by the European Commission for use as flavoring agents in food products. The concentrations were selected to be lower than those reported in the literature in order not to inhibit beneficial bacteria, based on previous laboratory experiments.

According to this protocol, the following experimental variants were established:

M1 = cabbage with the addition of 2.5% NaCl solution fermented at a temperature of 14 °C.

M2 = cabbage with the addition of 2.5% NaCl solution fermented at a temperature of 22 °C.

V1.1 = cabbage with the addition of 2.5% NaCl solution + 0.015% thyme essential oil fermented at a temperature of 14 °C.

V2.1 = cabbage with an added solution of 2.5% NaCl + 0.015% thyme essential oil fermented at a temperature of 22 °C.

V1.2 = cabbage with the addition of 2.5% NaCl solution + 0.015% dill essential oil, fermented at a temperature of 14 °C.

V2.2 = cabbage with an added solution of 2.5% NaCl + 0.015% dill essential oil, fermented at a temperature of 22 °C.

V1.3 = cabbage with the addition of 2.5% NaCl+ 0.015% wild thyme essential oil solution, fermented at a temperature of 14 °C.

V2.3 = cabbage with 2.5% NaCl solution added + 0.015% wild thyme essential oil, fermented at a temperature of 22 °C.

V1.4 = cabbage with the addition of 2.5% NaCl + 0.015 laurel essential oil, fermented at a temperature of 14 °C.

V2.4 = cabbage with the addition of 2.5% NaCl solution + 0.015% laurel essential oil, fermented at a temperature of 22 °C.

V1.5 = cabbage with the addition of 2.5% NaCl solution + 0.015% basil essential oil, fermented at a temperature of 14 °C.

V2.5 = cabbage with the addition of 2.5% NaCl solution + 0.015% basil essential oil, fermented at a temperature of 22 °C.

Every three days, sauerkraut was turned, which is an essential step in the traditional sauerkraut-making process, performed to ensure uniform fermentation, prevent spoilage (softening), and keep the brine clear. Fermentation was stopped by lowering the temperature to 8 °C to avoid entering the fourth fermentation phase when *Lactobacillus brevis* and other heterofermentative species capable of metabolizing pentoses, such as arabinose and xylose, become dominant and the finished product becomes altered. Subsequently, the fresh sauerkraut was stored in a refrigerator for 180 days at a temperature of 5 °C. For both the raw material (fresh white cabbage) and sauerkraut, immediately after lactic fermentation and sauerkraut’s removal from storage, pH, the total number of lactic acid bacteria, total soluble solid content (TSS%), ascorbic acid (mg·100 g fw), total sugar (g/100 g fw), total phenolics (GAE mg/100 g fw), and antioxidant activity (mmol Trolox/100 g fw) were determined. The fermentation duration was 12 days (until the pH remained constant).

### 2.5. Analytical Analysis

#### 2.5.1. pH Evaluation

pH was measured at an interval of three days using a Hanna Instruments laboratory pH meter (Nusfalau, Germany) with a pH electrode, −2 ÷ 20 pH.

#### 2.5.2. The Quantitative Determination of Lactic Acid Bacteria

The number of lactic acid bacteria was determined by inoculating 1 mL of each sample on MRS agar selective medium. To create anaerobic conditions, Genbag bags were used, in which a CO_2_-generating sachet was inserted. Bags with seeded plates were closed with a clip and thermostated at 30 °C for 3 days. The species-level identification of the LAB present in each experimental variant was not performed in the current study. Total colony counts on MRS agar provided an overall measure of LAB viability but did not distinguish between the different species that succeed one another during the four fermentation phases of sauerkraut. Characterization using 16S rRNA gene sequencing or shotgun metagenomics would be required to determine which specific taxa were differentially affected by the essential oil treatments. This constitutes a methodological limitation of the present work, and species-level microbiome analysis is identified as a priority objective for subsequent investigations.

#### 2.5.3. Evaluation of Total Soluble Solid Content

The total soluble solid content of the analyzed samples was determined using a digital refractometer (Euromex, Arnhem, The Netherlands) by measuring the angle of refraction of a light beam passing through the juice obtained from the sample to be analyzed at a temperature of 20 °C, and the results were expressed in percentages.

#### 2.5.4. Ascorbic Acid Evaluation

Ascorbic acid was quantified via RP-HPLC, utilizing a Finnigan Surveyor Plus platform (Thermo Electron Corporation, San Jose, CA, USA) coupled with a diode array detector. Sample preparation involved the dilution of 5 g of frozen cabbage homogenate to 100 mL using 0.1 N HCl. After a 30 min incubation period at room temperature, the mixture was centrifuged for 10 min at 4200 rpm. The resulting supernatant was then purified using a 0.2 μm filter. Analyte detection was set at 254 nm, with the final concentrations expressed in mg/100 g on a fresh weight (fw) basis, according to Nour et al. [26].

#### 2.5.5. Total Sugar Evaluation

The total sugar content was determined using the Dubois test, or phenol–sulfuric acid test, which involves a simple condensation reaction catalyzed by an acid and is commonly used to determine the total sugar concentration. The invert sugar extracted from the sample to be analyzed by boiling it with distilled water and inverting it with hydrochloric acid was neutralized with sodium hydroxide n/10. It was mixed in a vial with 1 mL of 5% (*w*/*v*) aqueous phenol solution, and 5 mL of concentrated sulfuric acid was then carefully added. The sample was left to rest for 10 min, shaken strongly, and incubated for another 30 min, after which the absorption of the extract at 490 nm was read using a Varian Cary 50 UV spectrophotometer (Varian Co., Palo Alto, CA, USA). The reaction mechanism is based on the formation of color by converting sugars into derivatives of furfurol with sulfuric acid. The amount of total sugar was determined using a calibration curve using different lactose concentrations as the standard [27]. The furfurol product was then condensed with phenol to produce stable-colored compounds.

#### 2.5.6. Total Phenolic Content Evaluation

To determine the total phenolic content and antioxidant capacity of the samples, methanol was used as the extraction solvent. Approximately 3 g of cabbage or sauerkraut was first homogenized, followed by extraction with 10 mL of methanol in an ultrasonic bath for 60 min at room temperature. After extraction, the samples were centrifuged at 6000 rpm for 15 min. The resulting supernatants were then separated and preserved at −40 °C until further analysis.

Total phenolic compounds were quantified using the Folin–Ciocalteu assay [28], with slight modifications. In brief, an aliquot of 100 μL of methanol extract was diluted with distilled water, followed by the addition of the Folin–Ciocalteu reagent. After a short reaction period, sodium carbonate solution (20% *w*/*v*) was introduced to promote color development, and the final volume was adjusted to 10 mL. The samples were mixed thoroughly and kept at 40 °C in the absence of light for 30 min. The intensity of the developed color was measured spectrophotometrically at 765 nm with a Varian Cary 50 UV spectrophotometer (Varian Co., Palo Alto, CA, USA). Quantification was achieved using a calibration curve constructed with gallic acid as the standard, and the results were expressed as mg gallic acid equivalents (GAE) per 100 g of fresh weight.

#### 2.5.7. Antioxidant Activity Evaluation

Antioxidant activity (AOA) was assessed using the procedure (free radical scavenging activity of extracts versus DPPH free radical) described by Nour et al. [26], and the results are expressed as μmol of Trolox equivalents per 100 g of fresh weight (fw).

Regarding all analytical analyses, each determination was carried out in triplicate, and the results presented correspond to the average of these repetitions.

### 2.6. Sensory Analysis

A preliminary sensory evaluation was performed on the finished product with the help of a group of 10 students (4 men and 6 women) from the Faculty of Horticulture who acted as semi-trained evaluators. Each evaluator scored the appearance, taste, smell, and aroma of the sauerkraut on a scale from 1 to 10 for each attribute, as well as the overall degree of acceptability of the product. The evaluation of sauerkraut was performed after 6 months of storage. Samples were coded randomly from 1 to 12. The coded samples were served to the panelists in plates containing both the sauerkraut and fermentation liquid. The panelists were then asked to evaluate only the former. After each tasting, the panelists were requested to eat bread and thoroughly rinse their mouths with water. A sensory analysis took place in the soundproof sensory analysis laboratory of the faculty at a temperature of 18–20 °C, with natural light and ventilation (air conditioning). Each panelist completed a scoring sheet, assigning hedonic (dislike very much, dislike, dislike a little, neither like nor dislike, like a little, like, or like very much) scores from 1 to 10 for each characteristic and the degree of acceptability of the product to provide a summative assessment of the attributes previously determined.

It is acknowledged that this panel size is below the threshold recommended by international sensory standards for consumer acceptability testing (n > 50 for untrained hedonic panels or 8–12 trained panelists for descriptive analysis methods such as Quantitative Descriptive Analysis [29]). This evaluation is therefore treated as a preliminary descriptive assessment intended to identify the most and least appreciated variants and guide formulation decisions, rather than a statistically validated consumer preference study. A full sensory study with an appropriately sized and screened panel is planned as part of the ongoing research program.

The assessment was carried out in accordance with international sensory analysis standards applicable to food testing: ISO 6658:2017 (SR ISO 6658)—General methods for planning sensory tests [30]; SR ISO 5492—Definitions and organoleptic terms; SR ISO 8589—Requirements for the sensory testing environment [31]; and the World Medical Association’s Declaration of Helsinki, which sets fundamental ethical principles for research involving human subjects.

To implement (EU) Regulation No 1169/2011 on the provision of food information to consumers and protect participants, we adopted the following measures.

Advance information: All participants received written notice prior to tasting about the presence of potentially allergenic or intolerance-causing ingredients.

Self-exclusion: Individuals who reported allergies to any ingredient in the tested product were excluded from participation.

Sample safety: Test samples were taken from batches deemed safe for human consumption and produced under food safety management systems (HACCP), thus minimizing any medical risk. The evaluation focused exclusively on product sensory characteristics rather than medical outcomes; therefore, review by a Clinical Ethics Committee was not mandatory. This study involved a technical product analysis conducted under safe, transparent conditions for volunteers. All panelists provided voluntary informed consent prior to participation. The declaration of compliance and safety for sensory analysis is in the Supplementary Materials Section.

### 2.7. Statistical Analysis

A statistical analysis was performed using Statgraphics Centurion XVIII software (StatPoint Technologies, Warrenton, VA, USA), and all tests were performed in triplicate. The results were presented as the mean ± standard deviation, and the least significant difference (LSD) test was used to estimate multiple pair-wise comparisons between means, with significant differences tested in an ANOVA (*p* < 0.05). In addition, Principal Component Analysis (PCA) was performed using the post-fermentation chemical data (ascorbic acid content, total phenolic content, antioxidant activity, and total soluble solids) across all experimental variants as a multivariate approach to explore the clustering of treatments and the correlations among chemical parameters, essential oil type, and fermentation temperature. PCA was conducted using Statgraphics Centurion XVIII (StatPoint Technologies, VA, USA) with all variables standardized to unit variance prior to analysis. This analysis was also performed using GraphPad Prism 10 (GraphPad Software, Boston, MA, USA) as a multivariate approach to explore the relationships among the chemical parameters measured across the experimental variants and to visualize the clustering of treatments according to essential oil type and fermentation temperature. The variables included in the PCA were ascorbic acid content, total phenolic content, antioxidant activity, and total soluble solids, which were all measured immediately after fermentation was stopped. All variables were standardized to unit variance prior to analysis. The results are presented as a biplot displaying both variable loadings and sample scores.

## 3. Results

### 3.1. pH Evaluation

**Figure 1:**
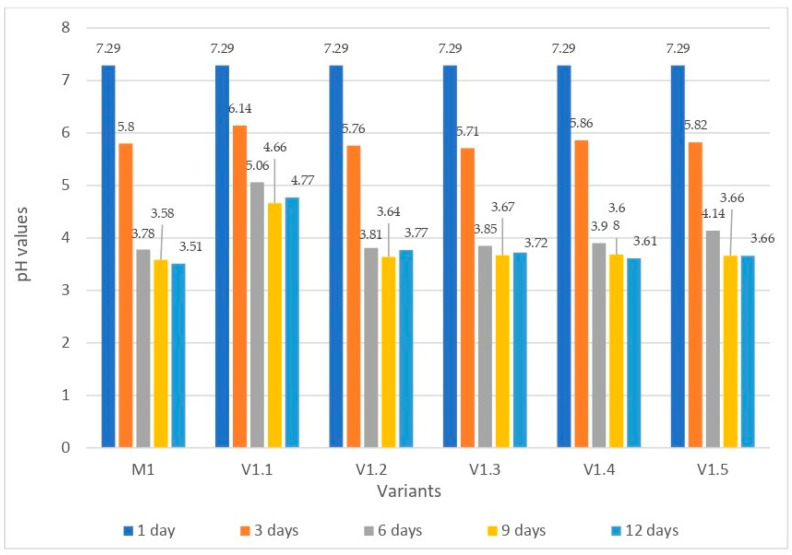
pH evaluation at a temperature of 14 °C.

**Figure 2:**
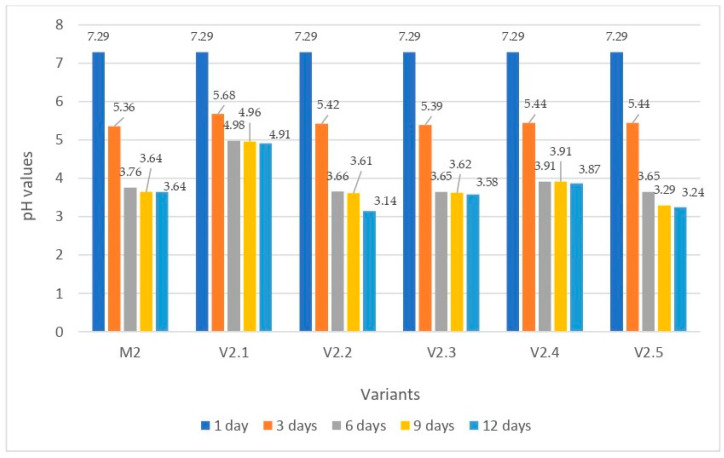
pH evaluation at a temperature of 22 °C.

The experimental variants were analyzed over 12 days, with the pH values recorded every 3 days. For each temperature examined (14 °C and 22 °C), control samples (without the addition of essential oil) were created and labeled as M1 and M2. The pH measurements in both the experimental variants and control samples indicated a more rapid decrease in pH during the initial days of fermentation for the variants fermented at 22 °C compared to those at 14 °C (Figure 1 and Figure 2). However, it was noted that at the end of fermentation, the pH values ranged from 3.14 (V2.2) to 4.91 (V2.1). Therefore, the lowest pH value was recorded in the variant treated with dill essential oil and fermented at 22 °C, while the highest value was found in the variant treated with thyme essential oil and fermented at 22 °C.

### 3.2. Evaluation of Number of Lactic Acid Bacteria

The total number of lactic acid bacteria was assessed after 6 and 12 days of fermentation. The lactic acid bacteria involved in cabbage fermentation exhibited population growth, with the highest counts being observed in the control variants (Table 1). This indicates that essential oils have an impact on these microorganisms. The data presented in the table below show that the number of lactic acid bacteria in both the control and experimental variants increased.

### 3.3. Evaluation of Main Chemical Components of Cabbage

The total soluble solid (TSS%) content of fresh cabbage was 5.5% (Table 2). Immediately after fermentation was stopped, the TSS content increased to values between 6.0 and 6.8%, with the highest ones being recorded in the V1.2 variant, followed by V1.1 and V2.4. Variants M1, V2.1, and V2.4 had the same TSS content value as V2.4.

During the 6-month storage of sauerkraut, the TSS content steadily decreased, reaching values between 4.0 (M2) and 4.8 (V1.2).

### 3.4. Total Sugar Content

The results regarding the total sugar content of sauerkraut are presented in Table 3. It is shown that the raw material (fresh cabbage) had an initial total sugar content of 3.34 g/100 g fw. Immediately after stopping fermentation, it was found that in the control samples, the quantity of residual sugar was recorded with values between 0.12 (M2) and 0.29 (M1), while in the other experimental variants, the total sugar content was undetected.

### 3.5. Ascorbic Acid Content

The variation in ascorbic acid content during lactic fermentation and the storage of sauerkraut is shown in Table 4.

The initial ascorbic acid content of fresh cabbage was 20.56 mg/100 g fw, which is comparatively lower than those found by Sarkar et al. [32] for various varieties of white cabbage from Bangladesh (37.23 mg/100 g fw). After 6 months of storage, the ascorbic acid content decreased significantly, with some variants showing values even lower than the initial content of the raw material (V1.3, V1.2, M1, M2, V2.4, and V1.1). However, there were significant differences between the analyzed variants in terms of the final ascorbic acid content when removing sauerkraut from storage. This content had values between 16.78 mg/100 g fw (V1.3—sauerkraut treated with wild thyme essential oil, fermented at 14 °C) and 29.62 mg/100 g fw (V2.2—sauerkraut treated with dill oil, fermented at 22 °C).

### 3.6. Total Phenolic Content

The total phenolic content was 18.69 mg GAE/100 g fw in the raw material (fresh white cabbage), which is a value much lower than those found by Sarkar et al. [32]. After lactic fermentation, it increased, with significant differences between the analyzed variants (Table 5).

The highest phenolic content was found after fermentation was stopped in variant V1.1. (29.62 mg GAE/100 g fw), followed by V1.2 (27.66 mg GAE/100 g fw) and V2.3 (25.32 mg GAE/100 g fw). While sauerkraut was stored in the refrigerator, a slight decrease in the total phenolic content was observed, thus maintaining the differences between the experimental variants. The increased levels of polyphenols and AOA may be due to the enzymatic activity of the cabbage’s native microflora, which can break down and release phenolic compounds from the cabbage’s cell walls [33]. Another explanation for this could be that the bacteria involved in fermentation can produce or even modify phenolic compounds [34].

### 3.7. Antioxidant Activity

The same trend was observed in terms of antioxidant activity (Table 6), which increased during lactic fermentation from 0.89 mmol Trolox/100 g fw in fresh cabbage to values ranging from 0.93 mmol Trolox/100 g fw (M1) to 1.96 mmol Trolox/100 g fw (V1.5). After 6 months of storage, a decrease in antioxidant activity was found with significant differences between the experimental variants. Thus, when sauerkraut was removed from storage, the lowest value of AOA was found in M1 (0.77 mmol Trolox/100 g fw) and the highest in V1.5 (1.79 mmol Trolox/100 g fw).

### 3.8. Principal Component Analysis

**Figure 3:**
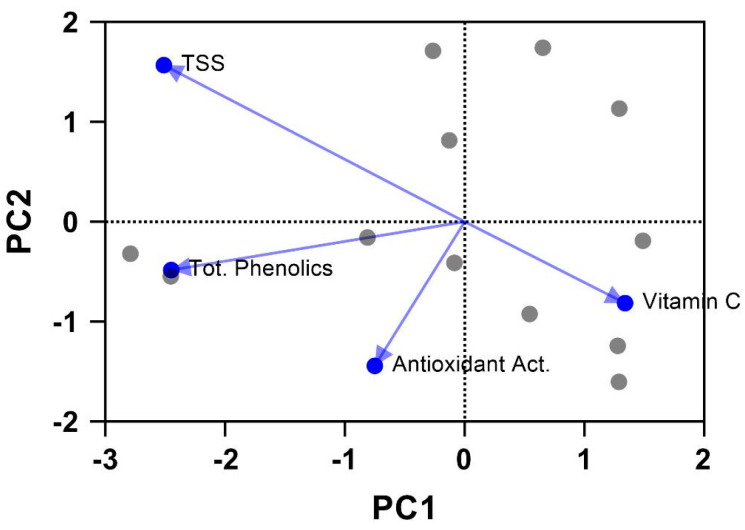
Principal component analysis.

To explore the overall relationships among the chemical parameters measured across all experimental variants, a Principal Component Analysis (PCA) was performed using ascorbic acid content, total phenolic content (TPC), antioxidant activity (AOA), and total soluble solids (TSS) as variables, which were measured immediately after fermentation was stopped. The first two principal components (PC1 and PC2) together explained 81.72% of the total variance (PC1: 51.02%; PC2: 30.70%), thus indicating that the chemical variability in the dataset is well captured in two dimensions (Figure 3).

The biplot in Figure 3 reveals a clear structure in the data. PC1 (51.02% of variance) was primarily driven by the opposing loadings of total phenolic content (negative direction, left quadrant) and ascorbic acid content (positive direction, right quadrant), thus indicating a negative correlation between these two variables across the experimental treatments. Variants treated with thyme essential oil (V1.1) and dill at 14 °C (V1.2), which exhibited the highest total phenolic content (29.62 and 27.66 mg GAE/100 g f.m., respectively), clustered in the negative PC1 region. In contrast, the control variants (M1 and M2) and those with high ascorbic acid content—particularly V2.2 (dill at 22 °C, 37.6 mg/100 g f.m.)—were positioned in the positive PC1 region. PC2 (30.70% of variance) separated variants according to TSS content (positive loading, upper quadrant) from those with high antioxidant activity (negative loading, lower quadrant). The control variants and thyme at 22 °C (V2.1), characterized by the lowest antioxidant activity values (0.93–0.98 mmol Trolox/100 g f.m.), clustered in the upper-right region, while variants with high antioxidant activity (V1.2, V1.3, and V1.5) occupied the lower quadrant of the biplot.

### 3.9. Sensory Analysis

**Figure 4:**
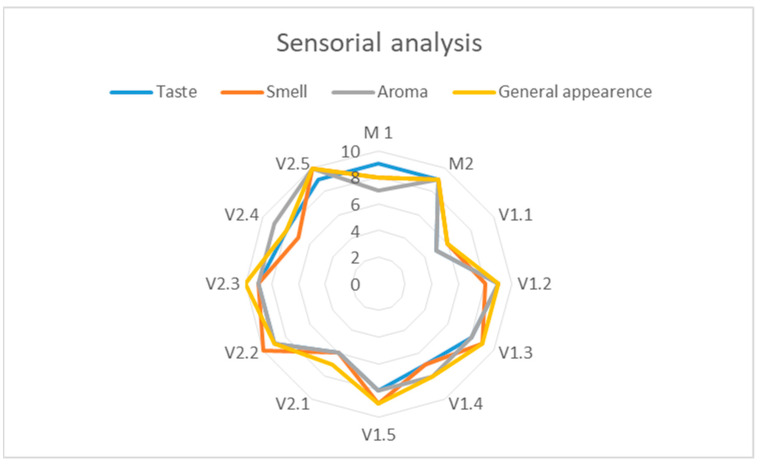
Sensory analysis.

The attributes evaluated in the sensory analysis included taste, aroma, smell, and general acceptability (Figure 4). In terms of taste, all variants, except those containing thyme essential oil, displayed a pleasant tangy flavor, with the oils being detectable in the aftertaste. The aroma was faintly perceptible from the essential oils, except for the thyme variants, in which the thyme scent was very strong and not well tolerated by the panelists. The smell predominantly featured sauerkraut in the top notes, while the oils were present in the mid-notes. The sensory analysis concluded that the most favored products were from variant V2.2 (sauerkraut with dill essential oil, fermented at 22 °C), followed by V2.5 (sauerkraut with basil essential oil, fermented at 22 °C) and V1.3 (sauerkraut with wild thyme essential oil, fermented at 14 °C). The sauerkraut with dill essential oil, fermented at 22 °C, was distinguished by its well-balanced flavor profile and aromatic qualities, thus making it appealing to the tasters. Close behind were variants V2.5 (sauerkraut with basil essential oil, fermented at 22 °C) and V1.5 (sauerkraut with basil essential oil, fermented at 14 °C). Variant V2.5 garnered positive feedback, which was likely due to the fresh and herbal notes of basil that complemented the sauerkraut’s acidity. Conversely, the version with thyme essential oil received criticism for its overpowering thyme taste and aroma. Considering this, it is recommended to conduct further experiments using lower concentrations of thyme essential oil.

## 4. Discussion

It must be acknowledged that in the present study, the chemical composition of the extracted essential oils was not characterized using Gas Chromatography–Mass Spectrometry (GC-MS) analysis. This represents a methodological limitation, as the identification of specific bioactive compounds (e.g., thymol and p-cymene in thyme, carvacrol and γ-terpinene in wild thyme, carvone and limonene in dill, eugenol in basil, and 1,8-cineole in laurel) would allow for a more mechanistic interpretation of the observed effects on lactic acid bacteria. However, based on published GC-MS profiles for essential oils extracted from the same plant species under hydrodistillation conditions [35,36,37], the dominant constituent classes are well established and discussed in the context of the microbiological results in Section 3.

During lactic fermentation, pH decreased concomitantly with the synthesis and accumulation of lactic acid in the fermentation mass due to the activity of lactic acid bacteria. It can be concluded that the added essential oil influenced the pH value during the fermentation of cabbage. From the data presented, regardless of the temperature applied, the pH decrease was shown to be steeper in the first phases of fermentation (3–6 days), after which it remained almost constant in the last ones. This corresponds to the first two fermentation phases, in which the most lactic acid accumulates in the fermentation mass. In the last days of fermentation, the pH value indicates the completion of the third stage of fermentation, which begins with another change in the lactic population; homofermentative lactobacilli become the predominant organisms, mainly due to the combined effect of anaerobiosis, low pH, and high salt levels. In the older literature, they are usually attributed to a single species, namely *Lactobacillus plantarum* (formerly called *Lactobacillus cucumeris*). The values obtained are in line with those in the literature, which state that most sauerkraut produced in Europe is pasteurized when it reaches a pH of 3.8 to 4.1, as consumers increasingly prefer light products, in terms of both acid content and salt [2].

Thyme essential oil (V1.1 and V2.1) inhibits the activity of the bacteria responsible for the fermentation of cabbage in the first stage, but as in the case of these experimental variants, there is an increase in the number of cells during fermentation. In contrast to the less inhibitory effect of dill, basil, and laurel essential oils, the differential inhibitory effect of thyme and wild thyme essential oils on LAB counts is consistent with the known chemical composition of these oils. Thyme (*Thymus vulgaris*) and wild thyme (*Thymus serpyllum*) essential oils are predominantly composed of phenolic monoterpenes—thymol and carvacrol—which exert potent antibacterial activity through the disruption of the bacterial cell membrane, dissipation of the proton motive force, and inhibition of ATPase activity [38,39]. By contrast, dill (*Anethum graveolens*) essential oil is rich in carvone and limonene [36], which are non-phenolic monoterpenes considerably less cytotoxic to LAB at low concentrations [40]. The concentration used in this study (0.015%) was below the minimum inhibitory concentration (MIC) reported in the literature for LAB species [40], which explains why fermentation was not fully inhibited in any variant. A complete GC-MS characterization of the oils used in this experiment is planned as a priority in a follow-up study.

The LAB community during sauerkraut fermentation follows a well-documented succession: the initial aerobic phase is dominated by heterofermentative species such as *Leuconostoc mesenteroides* and *Leuconostoc pseudomesenteroides*; as pH drops and anaerobic conditions are established, homofermentative species become dominant, including *Lactiplantibacillus plantarum* (formerly *Lactobacillus plantarum)*, thus driving fermentation to completion [21]. The phenolic monoterpenes present in thyme and wild thyme essential oils (primarily thymol and carvacrol) are known to be more inhibitory toward Gram-positive bacteria [38,39], which may preferentially suppress the early heterofermentative *Leuconostoc* populations without fully arresting the more acid-tolerant homofermentative *Lactiplantibacillus* species responsible for the terminal phase. This hypothesis is consistent with our observation that fermentation reached completion (near-zero residual sugars) in all variants, including those treated with thyme oil. However, species-level confirmation via 16S rRNA amplicon sequencing is necessary to validate this interpretation, and this is planned as part of a continuation of this research.

The decrease in the TSS content during storage can be attributed to the migration of soluble substances from fermented cabbage to cabbage juice. The increase in TSS content immediately after stopping lactic fermentation was also reported by Thakur et al. [41] with similar values. At the end of storage, the highest TSS contents were recorded for the variants treated with essential oils, namely V1.2 (cabbage treated with dill oil, fermented at a temperature of 14 °C) with a content of 4.8% and V1.1 (cabbage treated with thyme oil, fermented at a temperature of 14 °C) with a content of 4.6%. The fermentation temperature did not significantly influence the TSS content of the final product.

In almost all analyzed variants, total sugar content was not detectable at the end of fermentation. The total disappearance of sugar from sauerkraut at the end of fermentation was reported by Thakur et al. [41] and Zubaidah et al. [42].

At the end of fermentation, the measurable ascorbic acid content of sauerkraut was significantly higher than that of fresh cabbage in most variants, with values ranging from 22 mg/100 g f.m. (V1.2) to 37.6 mg/100 g f.m. (V2.2). The differences recorded for the same essential oil type (V1.2 vs. V2.2) demonstrate that fermentation temperature significantly influenced ascorbic acid retention and bioavailability in sauerkraut. Similar findings of higher measurable ascorbic acid content after lactic fermentation were reported by Thakur et al. [41]. The apparent increase in measurable ascorbic acid during fermentation is more accurately attributed to three complementary mechanisms, rather than representing de novo biosynthesis—which is not a metabolic capability of lactic acid bacteria. First, in Brassica vegetables, a substantial fraction of ascorbic acid exists in the form of ascorbigen, which is a condensation product of L-ascorbic acid and indole-3-carbinol formed from the indole glucosinolate glucobrassicin during fermentation [43,44,45]. Ascorbigen is not detected in the free ascorbic acid RP-HPLC fraction under standard conditions, but it releases measurable ascorbic acid progressively in an acidic fermentation environment (final pH 3.1–4.9 in the present study) [45]. Second, the anaerobic conditions and low pH established during lactic fermentation markedly reduce the oxidative degradation of ascorbic acid compared to aerobic processing, thus resulting in better net retention. Third, the consumption of soluble sugars (from 3.34 g/100 g f.m. in fresh cabbage to near-zero in EO variants) modifies the fresh matter matrix, which may alter the relative concentration of ascorbic acid expressed per 100 g of fresh matter. Collectively, these mechanisms explain the higher measurable ascorbic acid content in sauerkraut compared to fresh cabbage without invoking net biosynthesis.

The fermentation temperature did not significantly influence the total phenolic content or antioxidant activity of fresh sauerkraut. The results obtained are similar to those found by Chun et al. [46], Özer et al. [47], and Zhou et al. [48].

The antioxidant activity of cabbage increased during fermentation; however, in the absence of GC–MS analysis of the applied essential oils, it cannot be concluded that this increase is attributable to them. Essential oils are known to be rich sources of compounds with antioxidant properties, but they were used at very low concentrations. Consequently, their potential contribution to the observed enhancement in the antioxidant activity of sauerkraut cannot be reliably assessed. The fate of these compounds during fermentation, as well as their possible influence on the antioxidant properties of sauerkraut, requires further investigation. This limitation is acknowledged and will be addressed in future studies.

The PCA results provide a holistic view of the chemical modifications induced by different essential oils and fermentation temperatures. Accounting for 51.02% of total variance, PC1 is dominated by the opposing loadings of total phenolic content and ascorbic acid, thereby indicating that these two parameters are negatively correlated across treatments. This pattern reflects the distinct biochemical impact of phenolic monoterpene-rich oils (thyme and wild thyme)—which promote higher phenolic content in the final product—versus milder oils such as dill and basil, under whose influence ascorbic acid retention is favored. The partial separation of variants by temperature on PC2 (30.70%)—the most evident for the dill-treated samples (V1.2 vs. V2.2)—suggests that higher fermentation temperature (22 °C) accelerates ascorbigen hydrolysis and ascorbic acid liberation [41,43], thus resulting in higher measurable ascorbic acid in V2.2 compared to V1.2. The opposing positions of TSS and antioxidant activity on PC2 further indicate that variants with higher total soluble solid content tend to exhibit lower antioxidant activity, which is a relationship not apparent from univariate analysis. The two components together explain 81.72% of the total variance, thereby providing a robust two-dimensional characterization of the chemical variability in this dataset and confirming that essential oil identity is the primary driver of chemical differentiation among the experimental variants.

## 5. Conclusions

The addition of essential oils to cabbage before the onset of fermentation did not inhibit the development of lactic and acetic bacteria. However, it clearly influenced the growth of lactic acid bacteria and the chemical properties of the final product.

Dill essential oil appeared to provide the most promising results, combining efficient fermentation, a high ascorbic acid content, strong antioxidant activity, and the highest sensory acceptability.

The version obtained with the addition of thyme was completely rejected due to the pronounced taste and smell of thyme. Given this situation, this experiment should be resumed using lower concentrations of thyme essential oil.

The lactic fermentation of cabbage resulted in higher measurable ascorbic acid content compared to that in fresh raw material. This is attributed to the acid-catalyzed hydrolysis of ascorbigen—a bound, non-measurable ascorbic acid conjugate naturally present in Brassica tissue [41]—releasing free ascorbic acid during fermentation [43], combined with the protective effect of the anaerobic acidic environment against the oxidative degradation of ascorbic acid. Lactic fermentation leads to an increase in total phenolic content and antioxidant activity, while fermentation temperature does not influence these parameters.

This study lays the groundwork for further research into the analysis of volatile and phenolic compounds, as well as the influence of various essential oils on the lactic acid bacteria responsible for cabbage fermentation.

## Figures and Tables

**Table 1:** Total number of lactic acid bacteria during cabbage fermentation.

Variant	NTBL (UFC log) After 6 Days from the Start of Fermentation	NTBL (UFC log) 12 Days After the Start of Fermentation
M1	3.60 ± 0.16 ^eA^	6.47 ± 0.30 ^eB^
M2	3.04 ± 0.13 ^cA^	6.30 ± 0.29 ^deB^
V1.1	1.99 ± 0.08 ^aA^	4.06 ± 0.19 ^aB^
V1.2	2.54 ± 0.11 ^bA^	5.30 ± 0.24 ^bB^
V1.3	3.00 ± 0.12 ^cA^	6.30 ± 0.30 ^deB^
V1.4	3.36 ± 0.15 ^dA^	6.39 ± 0.29 ^eB^
V1.5	3.61 ± 0.17 ^eA^	6.50 ± 0.31 ^eB^
V2.1	1.98 ± 0.07 ^aA^	4.30 ± 0.19 ^aB^
V2.2	3.36 ± 0.14 ^dA^	5.91 ± 0.27 ^cdB^
V2.3	3.06 ± 0.13 ^cA^	5.60 ± 0.25 ^bcB^
V2.4	3.30 ± 0.14 ^dA^	5.90 ± 0.28 ^cdB^
V2.5	3.69 ± 0.18 ^eA^	5.87 ± 0.27 ^cdB^

The effects of the applied treatments on the number of total lactic acid bacteria during cabbage fermentation. Bars that share the same treatment and are marked with distinct lowercase letters show statistically significant differences, as determined using the least significant difference (LSD) test (*p* < 0.05). Similarly, bars that represent the same sampling time in different fermentation periods and are marked with different uppercase letters signify significant differences, as determined via the LSD test (*p* < 0.05).

**Table 2:** Variation in total soluble solid content (TSS%) in fresh cabbage, sauerkraut immediately after fermentation, and sauerkraut after 6 months of storage at temperature of 5 °C.

Variant	Fresh Cabbage	Sauerkraut Immediately After Fermentation	Sauerkraut After 6 Months of Storage
M1	5.5 ± 0.23 ^aB^	6.5 ± 0.31 ^abcC^	4.5 ± 0.19 ^bcdA^
M2	5.5 ± 0.23 ^aB^	6.0 ± 0.29 ^aC^	4.0 ± 0.17 ^aA^
V1.1	5.5 ± 0.23 ^aB^	6.6 ± 0.35 ^bcC^	4.6 ± 0.24 ^cdA^
V1.2	5.5 ± 0.23 ^aB^	6.8 ± 0.36 ^cC^	4.8 ± 0.22 ^dA^
V1.3	5.5 ± 0.23 ^aB^	6.4 ± 0.33 ^abcC^	4.4 ± 0.21 ^bcA^
V1.4	5.5 ± 0.23 ^aB^	6.4 ± 0.31 ^abcC^	4.4 ± 0.23 ^bcA^
V1.5	5.5 ± 0.23 ^aB^	6.2 ± 0.31 ^abC^	4.2 ± 0.22 ^abA^
V2.1	5.5 ± 0.23 ^aB^	6.5 ± 0.30 ^abcC^	4.5 ± 0.23 ^bcdA^
V2.2	5.5 ± 0.23 ^aB^	6.2 ± 0.32 ^abC^	4.2 ± 0.20 ^abA^
V2.3	5.5 ± 0.23 ^aB^	6.5 ± 0.33 ^abcC^	4.5 ± 0.23 ^bcdA^
V2.4	5.5 ± 0.23 ^aB^	6.5 ± 0.31 ^abcC^	4.5 ± 0.22 ^bcdA^
V2.5	5.5 ± 0.23 ^aB^	6.0 ± 0.27 ^aC^	4.0 ± 0.18 ^aA^

The variation in the total soluble solid content (TSS%) of fresh cabbage, sauerkraut immediately after fermentation, and sauerkraut after 6 months of storage at 5 °C. Bars that share the same treatment and are marked with different lowercase letters indicate statistically significant differences, as determined using the least significant difference (LSD) test (*p* < 0.05). Similarly, bars that represent the same sampling time at different fermentation and storage times and are marked with different uppercase letters signify significant differences, as determined via the LSD test (*p* < 0.05).

**Table 3:** Variation in total sugar content (g/100 g fw) in fresh cabbage, sauerkraut immediately after fermentation, and sauerkraut after 6 months of storage at temperature of 5 °C.

Variant	Fresh Cabbage	Sauerkraut Immediately After Fermentation	Sauerkraut After 6 Months of Storage
M1	3.34 ± 0.14 ^aB^	0.29 ± 0.01 ^bA^	-
M2	3.34 ± 0.14 ^aB^	0.12 ± 0.006 ^aA^	-
V1.1	3.34 ± 0.14 ^aA^	-	-
V1.2	3.34 ± 0.14 ^a^	-	-
V1.3	3.34 ± 0.14 ^a^	-	-
V1.4	3.34 ± 0.14 ^a^	-	-
V1.5	3.34 ± 0.14 ^a^	-	-
V2.1	3.34 ± 0.14 ^a^	-	-
V2.2	3.34 ± 0.14 ^a^	-	-
V2.3	3.34 ± 0.14 ^a^	-	-
V2.4	3.34 ± 0.14 ^a^	-	-
V2.5	3.34 ± 0.14 ^a^	-	-

The variation in the total soluble solid content (TSS%) of fresh cabbage, sauerkraut immediately after fermentation, and sauerkraut after 6 months of storage at 5 °C. Bars that share the same treatment and are marked with different lowercase letters indicate statistically significant differences, as determined using the least significant difference (LSD) test (*p* < 0.05). Similarly, bars that represent the same sampling time at different fermentation and storage times and are marked with different uppercase letters signify significant differences, as determined via the LSD test (*p* < 0.05).

**Table 4:** Variation in ascorbic acid content (mg/100 g fw) in fresh cabbage, sauerkraut immediately after fermentation, and sauerkraut after 6 months of storage at temperature of 5 °C.

Variant	Fresh Cabbage	Sauerkraut Immediately After Fermentation	Sauerkraut After 6 Months of Storage
M1	20.56 ± 1.07 ^aB^	28.8 ± 1.50 ^deC^	17. 6 ± 0.81 ^abA^
M2	20.56 ± 1.07 ^aB^	28.8 ± 1.39 ^deC^	18.2 ± 0.88 ^abA^
V1.1	20.56 ± 1.07 ^aB^	25.28 ± 1.23 ^bcC^	19.28 ± 0.94 ^bcA^
V1.2	20.56 ± 1.07 ^aB^	22 ± 0.98 ^aC^	17.4 ± 0.85 ^aA^
V1.3	20.56 ± 1.07 ^aB^	23.52 ± 1.15 ^abC^	16.78 ± 0.80 ^aA^
V1.4	20.56 ± 1.07 ^aB^	34.08 ± 1.64 ^ghC^	25.13 ± 1.17 ^eA^
V1.5	20.56 ± 1.07 ^aB^	35.84 ± 1.72 ^hiC^	27.43 ± 1.31 ^fA^
V2.1	20.56 ± 1.07 ^aB^	25.28 ± 1.27 ^bcC^	20.16 ± 0.96 ^cA^
V2.2	20.56 ± 1.07 ^aB^	37.6 ± 1.84 ^iC^	29.62 ± 1.46 ^gA^
V2.3	20.56 ± 1.07 ^aB^	32.32 ± 1.51 ^fgC^	26.98 ± 1.32 ^fA^
V2.4	20.56 ± 1.07 ^aB^	27.04 ± 1.33 ^cdC^	18.06 ± 0.87 ^abA^
V2.5	20.56 ± 1.07 ^aB^	30.56 ± 1.49 ^efC^	23.71 ± 1.11 ^eAB^

The variation in the ascorbic acid (mg/100 g fw) content of fresh cabbage, sauerkraut immediately after fermentation, and sauerkraut after 6 months of storage at 5 °C. Bars that share the same treatment and are marked with different lowercase letters indicate statistically significant differences, as determined using the least significant difference (LSD) test (*p* < 0.05). Similarly, bars that represent the same sampling time at different fermentation and storage times and are marked with different uppercase letters signify significant differences, as determined via the LSD test (*p* < 0.05).

**Table 5:** Variation in total phenolic content (GAE mg/100 g fw) in fresh cabbage, sauerkraut immediately after fermentation, and sauerkraut after 6 months of storage at a temperature of 5 °C.

Variant	Fresh Cabbage	Sauerkraut Immediately After Fermentation	Sauerkraut After 6 Months of Storage
M1	18.69 ± 0.90 ^aA^	22.71 ± 1.10 ^aB^	20.64 ± 1.00 ^abA^
M2	18.69 ± 0.90 ^aA^	24.57 ± 1.19 ^abC^	21.19 ± 1.03 ^abcB^
V1.1	18.69 ± 0.90 ^aA^	29.62 ± 1.51 ^bC^	25.79 ± 1.22 ^dB^
V1.2	18.69 ± 0.90 ^aA^	27.66 ± 1.36 ^bC^	22.47 ± 1.06 ^cB^
V1.3	18.69 ± 0.90 ^aA^	25.04 ± 1.22 ^bC^	21.52 ± 0.97 ^bcB^
V1.4	18.69 ± 0.90 ^aA^	24.39 ± 1.17 ^abB^	20.26 ± 0.93 ^abA^
V1.5	18.69 ± 0.90 ^aA^	23.83 ± 1.15 ^abB^	19.61 ± 0.91 ^aA^
V2.1	18.69 ± 0.90 ^aA^	24.39 ± 1.18 ^abC^	21.37 ± 1.01 ^bcB^
V2.2	18.69 ± 0.90 ^aA^	24.95 ± 1.26 ^bC^	21.39 ± 1.03 ^bcB^
V2.3	18.69 ± 0.90 ^aA^	25.32 ± 1.31 ^bC^	22.51 ± 1.16 ^cB^
V2.4	18.69 ± 0.90 ^aA^	24.01 ± 1.24 ^abC^	20.83 ± 0.99 ^abcB^
V2.5	18.69 ± 0.90 ^aA^	23.64 ± 1.15 ^abB^	19.94 ± 0.87 ^abA^

The variation in the total phenolic content (TP mg GAE/100 g fw) of fresh cabbage, sauerkraut immediately after fermentation, and sauerkraut after 6 months of storage at 5 °C. Bars that share the same treatment and are marked with different lowercase letters indicate statistically significant differences, as determined using the least significant difference (LSD) test (*p* < 0.05). Similarly, bars that represent the same sampling time at different fermentation and storage times and are marked with different uppercase letters signify significant differences, as determined via the LSD test (*p* < 0.05).

**Table 6:** Variation in antioxidant activity (mmol Trolox/100 g fw) in fresh cabbage, sauerkraut immediately after fermentation, and sauerkraut after 6 months of storage at temperature of 5 °C.

Variant	Fresh Cabbage	Sauerkraut Immediately After Fermentation	Sauerkraut After 6 Months of Storage
M1	0.89 ± 0.03 ^aB^	0.93 ± 0.04 ^aB^	0.77 ± 0.02 ^aA^
M2	0.89 ± 0.03 ^aA^	0.98 ± 0.05 ^aB^	0.81 ± 0.04 ^aA^
V1.1	0.89 ± 0.03 ^aA^	1.64 ± 0.07 ^cdeC^	1.12 ± 0.05 ^cB^
V1.2	0.89 ± 0.03 ^aA^	1.84 ± 0.09 ^fC^	1.67 ± 0.06 ^eB^
V1.3	0.89 ± 0.03 ^aA^	1.80 ± 0.09 ^fC^	1.59 ± 0.07 ^eB^
V1.4	0.89 ± 0.03 ^aA^	1.74 ± 0.07 ^efC^	1.23 ± 0.05 ^dB^
V1.5	0.89 ± 0.03 ^aA^	1.96 ± 0.08 ^gC^	1.79 ± 0.09 ^fB^
V2.1	0.89 ± 0.03 ^aA^	0.98 ± 0.03 ^aC^	0.82 ± 0.03 ^aB^
V2.2	0.89 ± 0.03 ^aA^	1.66 ± 0.06 ^deC^	1.11 ± 0.04 ^cB^
V2.3	0.89 ± 0.03 ^aA^	1.55 ± 0.07 ^cdC^	1.05 ± 0.05 ^bcB^
V2.4	0.89 ± 0.03 ^aA^	1.32 ± 0.05 ^bC^	0.97 ± 0.03 ^bB^
V2.5	0.89 ± 0.03 ^aA^	1.54 ± 0.06 ^cC^	1.09 ± 0.05 ^cB^

The variation in the radical scavenging activity (AOA mmol Trolox/100 g fw) content of fresh cabbage, sauerkraut immediately after fermentation, and sauerkraut after 6 months of storage at 5 °C. Bars that share the same treatment and are marked with different lowercase letters indicate statistically significant differences, as determined using the least significant difference (LSD) test (*p* < 0.05). Similarly, bars that represent the same sampling time at different fermentation and storage times and are marked with different uppercase letters signify significant differences, as determined via the LSD test (*p* < 0.05).

## Data Availability

The original contributions presented in this study are included in the article/Supplementary Materials. Further inquiries can be directed to the corresponding author.

## Supplementary Materials

The following supporting information can be downloaded at: https://www.mdpi.com/article/10.3390/foods15152746/s1, S1: Declaration of compliance and safety for sensory analysis.

## List of Abbreviations

DM	dry matter content
TSS	total soluble solid content
GAE	gallic acid equivalent
NTBL	number of lactic acid bacteria
AOA	antioxidant activity
MIC	minimum inhibitory concentration
fw	fresh weight
