# Supplementary material for: The Influence of Incorporating Essential Plant Oils in the Lactic Fermentation Process of Cabbage on the Quality of Sauerkraut
Source: Foods. 2026 Aug 5;15(15):2746. doi: 10.3390/foods15152746 (PMC13465722; doi:10.3390/foods15152746)
Supplement: Supplementary file 1 [file foods-15-02746-s001.zip › foods-4387234-supplementary.pdf]

UNIVERSITY OF CRAIOVA

FACULTI OF HORTICULTURE

DECLARATION OF COMPLIANCE AND SAFETY FOR SENSORY ANALYSIS

I, Felicia Tuțulescu, in my capacity as a university professor, hereby declare under my own responsibility that the methodology applied in this sensory analysis study (*Modifications during sauerkraut processing induced by adding plant-derived essential oils*), complies with the legal and technical framework in Romania and adheres to the following safety and ethical principles:

Technical Basis (ISO/ASRO standards)

The assessment was conducted in accordance with international sensory analysis standards that govern food testing procedures:

• ISO 6658:2017 (SR ISO 6658) — General methodology for planning sensory tests;

• SR ISO 5492 — Definitions and organoleptic terms;

• SR ISO 8589 — Requirements related to the testing environment.

Safety Protocol and Allergen Management

In implementing Regulation (EU) No 1169/2011 on the provision of food information to consumers, I have adopted the following measures to protect participants:

• Prior information: Each participant receives written information before tasting about the presence of any potentially allergenic or intolerance-causing ingredients (according to the official list: gluten, lactose, peanuts, etc.);

• Self-exclusion: Persons who declare allergies to any of the product's ingredients are excluded from tasting;

• Sample integrity: Tested products originate from batches safe for human consumption and comply with food safety standards (HACCP), thereby reducing any medical risk.

Ethical Status and Confidentiality (GDPR)

• Non-clinical nature: The testing targets only product characteristics, not complex medical reactions, and therefore approval from a Clinical Ethics Committee is not required;

• Data anonymization: Data are collected using coding so that responses cannot be linked to participants' identities. Informed consent covers voluntary participation in tasting and acknowledgement of the ingredient list.

This declaration confirms that the study constitutes a technical procedure of product analysis, carried out under safe and transparent conditions for volunteers.

Date: 11.11.2025

Signature:
